# Environmental Implications of Ionic Liquid and Deep Eutectic Solvent in Geothermal Application: Comparing Traditional and New Approach Methods

**DOI:** 10.1021/acssuschemeng.4c04606

**Published:** 2024-09-23

**Authors:** Amin Atashnezhad, Justin Scott, Mohammed F. Al Dushaishi

**Affiliations:** † School of Chemical Engineering, Oklahoma State University, Stillwater, Oklahoma 74078, United States; ‡ Cove Environmental LLC, Stillwater, Oklahoma 74075, United States; § School of Civil and Environmental Engineering, Oklahoma State University, Stillwater, Oklahoma 74078, United States

**Keywords:** Pyridinium-based ionic liquid, Magnesium chloride hexahydrate, Choline chloride, Hexylepyradinium bromide, Alternative toxicity tests, In vitro toxicity, In vivo assays, Geothermal fluids toxicity

## Abstract

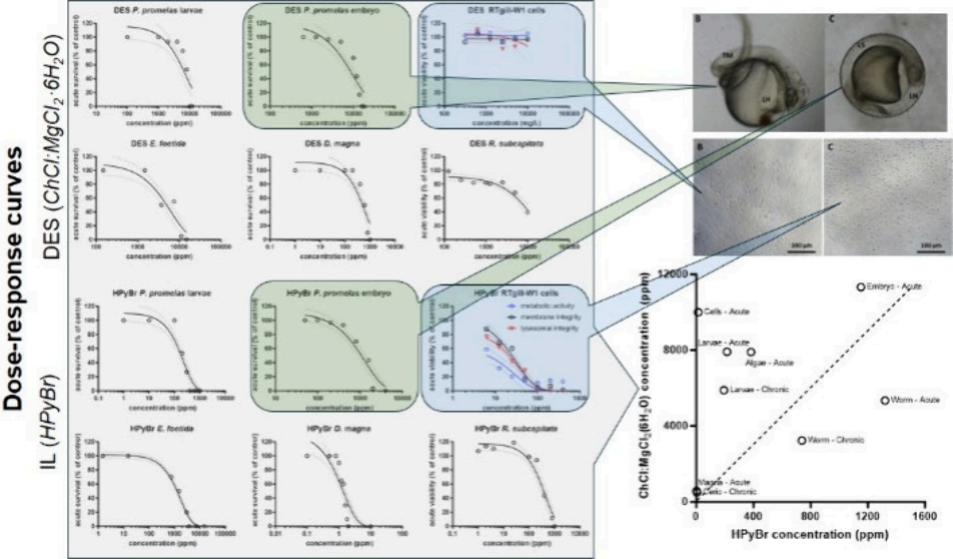

The significant surge of ionic liquids (ILs) research over the past decade has led to the formation of various novel ionic liquid compounds and their diverse applications. Enhanced geothermal systems (EGS) for geothermal power generation are an emerging IL application as a heat extraction fluid. The once widely held belief in the environmentally friendly characteristics of ionic liquids, mainly due to their insignificant vapor pressure, is now being scrutinized. It has become apparent that while ILs do not readily evaporate into the atmosphere, they are not guaranteed to remain entirely isolated from the environment. Recent attention has been directed toward toxicological studies, including ecotoxicity impacts, with the long-accepted assumption of ILs having low toxicity being invalid. This paper aims to shed light on the toxicity of hexylepyradinium bromide (HPyBr) IL and a deep eutectic solvent (DES) comprising choline chloride with magnesium chloride hexahydrate (ChCl:MgCl_2_·6H_2_O) to five test species, an algal species (*Raphidocelis subcapitata*), the water flea (*Ceriodaphnia dubia and Daphina magna)*, the fathead minnow (*Pimephales promelas*), and the earthworm (*Eisenia fetida*), to measure acute and chronic toxicity. Additionally, new approach methods (NAMs) are presented using the fathead minnow embryo and the rainbow trout (*Oncorhynchus mykiss*) gill cell line and the RTgill-W1 assay to compare sensitivity across species. Overall, ChCl:MgCl_2_·6H_2_O displayed lower toxicity, while HPyBr demonstrated higher toxicity, highlighting the need for caution in handling it to prevent harm to aquatic ecosystems. Comparative analysis underscored the potential threat of ChCl:MgCl_2_·6H_2_O to aquatic life, highlighting the cumulative effects of the environmental components.

## Abstract 2

Toxicity evaluation of ionic liquid and DES to various test species for sustainable geothermal energy.

## 1 Introduction

Ionic liquids (ILs) are a different category of solvents considered by their salt-like structure, usually containing organic cations (including ammonium, imidazolium, pyridinium, piperidinium, or pyrrolidinium) and various anions, such as halogen, fluorinated, or organic variants.^1^ They are known for their low melting points, made possible by the substantial size and asymmetry of the ions involved.^2^ ILs can be found in various applications, serving as multipurpose reaction media for organic and biochemical synthesis, solutions for efficient metal extraction, electrolytes in batteries, facilitators in polymeric electrolyte membrane fuel cells, components in solar cells, contributors to many biological applications, and essential agents in biomass processing applications.^3^ They serve well as a novel medium for liquid–liquid extractions of metal ions using organic metal chelators, where ILs as a liquid–liquid extraction medium showed an extraction efficiency of 98.4%. When utilized as a chelate extraction solvent, ILs show drastically higher extraction capabilities than their organic counterparts.^3^ Ionic liquids are found in modern electronics and batteries,^4,5^ where ILs are a safer option than conventional liquid electrolyte batteries.^5^ As proposed by Shin et al.^5^ room-temperature ILs showcase high conductivity, making them explicitly promising for increasing battery performance in portable electronic devices operating at approximately 25 °C.

Due to their unique properties, ionic liquids have gained attraction as potential heat transfer and working fluids in geothermal systems. ILs offer several advantages over traditional working fluids, including high thermal stability and tunability. These advantages could make them a promising option for enhancing the efficiency and sustainability of geothermal energy production.

Ionic liquids have higher thermal stability than water-based fluids, making them more suitable for geothermal energy systems. A thermal stability investigation of 66 distinct ILs revealed that approximately 50% of the analyzed ILs have a thermal stability exceeding 300 °C, with 25% surpassing 400 °C, indicating exceptional thermal stability for some ILs.^6^ Compared to typical heat transfer fluids, the thermal conductivity and heat storage density of various ILs showed promising characteristics that make them suitable for heat transfer fluids.^7^ Several investigations addressed ILs as potential geothermal working fluids.^8−11^ Kazemi et al.^8^ investigated the use of IL as a geothermal working fluid with an organic Rankine cycle by evaluating the energy extraction efficiency. Atashnezhad et al.^9^ studied the rheological behavior of two ILs under high pressure and high temperature for geothermal application to enhance fluid flow in fractured geothermal reservoirs. Momoh et al.^10^ addressed the effect of ILs on fracture conductive and reversibility behavior, i.e., reservoir damage. Their work showed that IL reversibility increases with the temperature. Previous investigations addressing ILs in geothermal applications neglected the environmental impact. Environmental impact and toxicity must be evaluated for full-scale testing and field implementation.

While ILs have been identified as environmentally friendly substances, the evidence supporting this statement is limited. Their significant features include high thermal stability and low volatility at room temperature, which avoid the release of ILs into the atmosphere. Nonetheless, the increasing adoption of ILs across many chemical industries has triggered concerns about potential pollution of aquatic and terrestrial environments. New analogs of ILs, such as deep eutectic solvents (DESs), have gained momentum for their applications and warrant investigation for their environmental impact.^12^ Importantly, as the chances of systematic release of ILs into the environment continue to grow, there is a need to evaluate their ecotoxicological impacts. Many bioassays measured the ecotoxicity and biodegradability of ILs, and many of these studies have raised questions about their green credentials, as ILs have exhibited toxicity toward organisms at different trophic levels.^1^ ILs and DESs have disadvantages that make them less green than previously claimed, such as decomposition, volatility, toxicity, and flammability.^13^ ILs have higher volatility, flammability, and explosibility compared to DESs, however, these issues are only observed under extreme conditions.^13^ Therefore, there is an increasing demand for developing biodegradable ILs that are less toxic to aquatic ecosystems.

The aquatic toxicity of ILs has been evaluated in several investigations using different approaches. For instance, Ranke et al.^14^ performed bacteria-based assays of imidazolium ILs in acute *Vibrio fischeri* and WST-1 cells. Montalbán et al.^15^ investigated the effect of the anion and the side chain in the cation of different imidazolium-, pyridinium- and ammonium-based ILs toxicity to bioluminescent photobacterium *Vibrio fischeri*. While the cell-based assay with *Vibrio fischeri* was most widely used to assist aquatic toxicity of ILs, different organisms have been used. Pham et al.^16^ used photosynthetic assays with alga Pseudokirchneriella subcapitata to assist the toxicity of imidazolium- and pyridinium-based ILs. Different aquatic organisms that have been used to evaluate the toxicity of ILs include aquatic plants such as duckweed Lemna minor,^17,18^ invertebrates such as *Daphnia magna*,^19^ and vertebrates such as zebrafish/Danio rerio.^19,20^ Toxicity studies of *n*-hexyl-pyridinium bromide (HPyBr) IL and choline chloride with magnesium chloride hexahydrate (ChCl:MgCl2·6H2O), addressed in this paper, are limited, and further investigations are required. For instance, Chen and Mu discussed the greenness of 64 ILs and DESs, where HPyBr and ChCl:MgCl_2_·6H_2_O were not evaluated.^13^

Current toxicity assays primarily utilize in vivo aquatic and terrestrial model organisms. Specifically, for chemicals such as ILs that are highly soluble in water, aquatic invertebrate and vertebrate organisms such as freshwater algae (*Raphidocelis subcapitata*), daphnids (*Ceriodaphnia dubia* and *Daphnia magna*), and fish (*Pimephales promelas*) serve as good models for assessing acute and chronic toxicity end points.^21,22^ All three aquatic toxicity assays have been standardized to allow laboratory repeatability and reproducibility to various acute and chronic exposure scenarios, such as individual chemical assessments for registration and ambient, wastewater, and receiving streams. Specifically, acute mortality can be coupled with algal proliferation, daphnid neonate reproduction, and fish growth measured upon chronic exposures. Additionally, where ILs commercial applications, such as geothermal applications,^8−11^ may allow interaction with soil, the earthworm (*Eisenia fetida*) toxicity assay looking at mortality and growth can also provide toxicological information for acute,^23^ and chronic exposures,^24^ respectively.

While the previously mentioned assays offer an established approach to measuring toxicity, new approach methods (NAMs) such as the fish embryo acute toxicity assay^25^ and the rainbow trout (*Oncorhynchus mykiss*) fish gill cell line, RTgill-W1, based assay^26^ offer a more informative and ethical approach. Previous studies have highlighted the use of these NAMs for predicting toxicity at the organismal level.^27−29^ FET assays predict lethality in larvae through the observations of developmental markers related to teratogenicity. This approach allows more toxicological effects to be measured at an essential stage in the development of the fish life cycle. RTgill-W1 cells are similar, if not more sensitive, to fish to common and emerging contaminants of concern.^30^ Moreover, cell-based cytotoxicity assays offer a better understanding of the toxic mechanism of action through subcellular viability markers and serve as a proper replacement for in vivo assays.

To build on this concept, the main objectives of this study are (i) determine the acute and chronic toxicity of ILs using current toxicity models and (ii) evaluate if alternative embryo and cell models can provide additional toxicological information for IL applications. Overall, the study highlighted the differences between ILs and their toxicity related to the species of concern. Furthermore, it has been shown that the use of NAMs can provide additional information about the toxicity of ILs that current toxicity testing methods do not provide.

## 2 Methodology

### 2.1 Chemical Synthetization and Exposure Solution Preparation

The chemical components used to synthesize the DES and IL were purchased from Fisher Scientific International, Inc. The DES chemicals comprised 99% pure Choline Chloride manufactured by Thermo Scientific Chemicals and ≥99.0% pure Magnesium Chloride Hexahydrate manufactured by Fisher BioReagents. The raw materials for HPyBr IL consisted of 98.0+% pure 1-bromobutane manufactured by TCI America and 99+% extra pure pyridine manufactured by Thermo Scientific Chemicals.

The deep eutectic solvent (DES) of ChCl:MgCl_2_·6H_2_O was diluted during synthetization. This practice of diluting DES with water has been previously explored in the literature.^31^ López-Salas et al.^31^ highlighted the advantages of diluting DES in water, which leads to a distortion of the tetrahedral structure and the formation of new eutectics with lowered melting points, presenting potential benefits such as reduced viscosity and cost-effectiveness. The diluted ChCl:MgCl_2_·6H_2_O consisted of a 1.7 mol ChCl, 1 mol MgCl_2_·6H_2_O, and 1 mol distilled water. Solid choline chloride (118.66 g) and solid magnesium chloride hexahydrate (101.5 g) were placed in a 500 mL glass beaker with 9 mL of water and stirred at room temperature.

The synthesis of N-hexyl-pyridinium bromide (HPyBr) IL consisted of adding 1-bromobutane (0.52 mol, 85.8 g, 72.7 cc) dropwise to pyridine (0.5 mol, 39.5 g, 40.3 cc) in a two-neck round-bottom flask fitted with a reflux condenser and a drying tube. The mixture was stirred at room temperature for 24 h and then stirred at 60 °C for another 24 h. The viscous product was washed two times with ethyl acetate. The resulting N-hexylpyridinium bromide was vacuum-dried at 60 °C to remove the remaining ethyl acetate and excess 1-bromoethane.

The aquatic toxicity studies included daphnids, fish, and algae species, and the terrestrial toxicity studies included earthworm species (Table 1). USEPA moderately hard water was spiked with the ILs and mixed before exposure.^22^ Soil toxicity studies using the earthworm utilized the OECD and USEPA synthetic soil scenarios spiked with the ILs and mixed before exposure. For cytotoxicity assays using RTgill-W1 cells, an exposure media mimicking Leibovitz’s L-15 media, but only including salts and sugars sodium pyruvate and galactose, spiked with the ILs and mixed before exposure. Soil exposure media conditions consisted of a dry weight basis of 10% sphagnum peat moss, 20% kaolin clay, and 70% silica sand. The exposure concentrations for each IL ranged from HPyBr, 0.625–14925 ppm, and DES, 1–14925 ppm.

**Table 1:** Model Organisms Used in Aquatic Toxicity Assessment

Exposure Type	Species
Acute	*Daphnia magna*
	*Oncorhynchus mykiss* - cells
	*Pimephales promelas* - larvae
	*Raphidocelis subcapitata*
	*Pimephales promelas* - embryo
	*Eisenia fetida*
	
Chronic	*Ceriodaphnia dubia*
	*Pimephales promelas* - larvae
	*Eisenia fetida*

### 2.2 Culturing of Organisms and Toxicity Assays

All in vivo organisms were obtained from Cove Environmental’s aquatic toxicity laboratory (Stillwater, OK) and cultured from spawning broodstock following various culturing guidelines. RTgill-W1 cells were purchased from the ATCC (CRL-2523). Algae (*R. subcapitata*), both daphnids (*C. dubia* and *D. magna*), and fathead minnow (*P. promelas*) embryos and larvae were cultured following USEPA guidelines.^21,22^ Earthworms (*Eisenia fetida*) were cultured following OECD guidelines,^23^ and RTgill-W1 cells were cultured following OECD guidelines.^26^ Aquatic in vivo toxicity assay parameters and end points followed established toxicity testing guidelines.^21,22,25^ Soil toxicity assay parameters and end points followed established toxicity testing guidelines.^23,24^ RTgill-W1 cell assays followed established cytotoxicity testing guidelines.^26^ Briefly, the methods for the toxicity assays are described below.

*R. subcapitata* chronic toxicity assays utilized cells ranging between four and 7 days old and were inoculated at 10,000 cells/mL in 50 mL working volume (four replicates per concentration) at a continuous shaking rate of 100 cpm on an orbital shaker. Assays were conducted at 25 ± 1 °C with a light intensity of 400 ft-c and a photoperiod of continuous illumination. Exposure duration consisted of 96-h, with algal cell growth being measured as absorbance (Hach DR3900 Benchtop Spectrophotometer) to determine the effect concentration of 50% of the population (EC50) and the no observable effect concentration (NOEC).

*C. dubia* chronic toxicity assays utilized neonates less than 24-h old and contained one organism per container of 15 mL working volume (10 replicates per concentration). Organisms were fed daily with 0.1 mL of 1:1 algae (*R. subcapitata*) and yeast, cereal, and trout chow (YCT) mixture based on USEPA’s recipe (USEPA, 2002b). Assays were conducted at 25 ± 1 °C with a light intensity of 50–100 ft-c and a photoperiod of 16:8, light: dark. Exposure duration consisted of 7 days with the end points of female survival for the lethal concentration of 50% population (LC50) and young brood counts being measured daily to determine the reproduction NOEC.

*D. magna* acute toxicity assays *C. dubia* chronic toxicity assays utilized neonates under 24-h old and contained eight organisms per container of 30 mL working volume (5 replicates per concentration). Daily, Organisms were fed 0.1 mL of 1:1 algae (*R. subcapitata*) and YCT. Assays were conducted at 25 ± 1 °C with a light intensity of 50–100 ft-c and a photoperiod of 16:8, light: dark. The exposure duration was 96 h, with survival measured daily to determine the LC50.

*P. promelas* larvae chronic toxicity assays utilized less than 24-h old larvae and contained ten organisms per container of 250 mL working volume (3 replicates per concentration). The larvae were fed twice daily with 24-h old *Artemia nauplii*. Assays were conducted at 25 ± 1 °C with a light intensity of 50–100 ft-c and a photoperiod of 16:8, light: dark. The exposure duration consisted of 7 days, with larvae’s survival measured daily to determine the LC50 and biomass measured at test termination to determine the growth of NOEC.

*P. promelas* larvae acute toxicity assays utilized 14-d old larvae and contained ten organisms per container of 250 mL working volume (3 replicates per concentration). The larvae were not fed during the test duration. Assays were conducted at 25 ± 1 °C with a light intensity of 50–100 ft-c and a photoperiod of 16:8, light:dark. The exposure duration consisted of 7-d, with larvae’ survival measured daily to determine the LC50 and biomass measured at test termination to determine the growth of NOEC.

*P. promelas* FETs acute toxicity assays utilized the ≤32-cell stage. The embryos were placed into the well of a 24-well plate (one egg per well, 20 eggs per concentration) containing 2.5 mL of exposure solution. Measured toxicity end points consisted of (i) coagulation of the embryos, (ii) lack of somite formation, (iii) nondetachment of the tail, and (iv) lack of heartbeat to determine the LC50.

*E. fetida* acute and chronic toxicity assays utilized adult earthworms weighing between 300 and 600 mg. Based on the EPA’s recipe, earthworms were placed into 1 L glass containers with lids containing artificial soil. Assays were conducted at 22 ± 2 °C with a light intensity of 40–50 ft-c and a continuous photoperiod left on. The exposure duration consisted of 28 days, with measured toxicity end points consisting of mortality to determine the LC50 and biomass to determine the growth of NOEC.

*E. fetida* acute toxicity assays utilized adult earthworms weighing between 300 and 600 mg. Based on the EPA’s recipe, earthworms were placed into 1 L glass containers with lids containing artificial soil. Assays were conducted at 22 ± 2 °C with a light intensity of 40–50 ft-c and a continuous photoperiod left on. Exposure duration consisted of 14 days for acute and 28 days for chronic, with measured toxicity end points consisting of mortality to determine the LC50 and biomass to determine the growth NOEC.

RTgill-W1 cells (DSMZ ACC No. 899) acute toxicity assays utilized cells seeded at a density of 150,000 cells/mL (78,947 cells/cm^2^) into 24 multiwell plates (Greiner Bio-One, Kremsmunster). All plates were then incubated at 19 °C in the dark for 48 h to allow for the development of a confluent monolayer. Cells were then exposed and incubated at 19 °C in darkness for 24 h. After 24-h of exposure, cells were rewashed, and the multiple end point viability assays, which measure cell metabolic activity, membrane integrity, and lysosomal integrity through the application of three commercially available dyes, which are alamarBlue (AB; Invitrogen, Thermofisher, Waltham, MA, USA), 5-carboxyfluorescein diacetate acetoxymethyl ester (CFDA-AM; Thermofisher, Waltham, MA, USA), and 3-amino-7-dimethylamino-2-methylphenazine hydrochloride or Neutral Red (NR; Sigma-Aldrich, St. Louis, MO, USA), respectively, were performed to determine the EC50.^26.32^

### 2.3 Statistical Analysis

The effective concentration of 50% (EC50) and lethal concentration of 50% (LC50) were calculated based on mortality or viability reported as a percent of negative control using the nonlinear regression sigmoidal dose–response curve fitted module using the Hill slope equation. A two-tailed Pearson’s r coefficient test was performed for correlation data. All experiments were repeated at least three times for each sample. All statistical analysis tests were checked for normality and performed with an alpha value equal to 0.05 using GraphPad Software (Prism version 9.4). All data for EC50 and LC50 correlations were found to be normally distributed through D’Agostino and Pearson tests, and data was run using parametric analysis.

## 3 Results and Discussion

**Figure 1:**
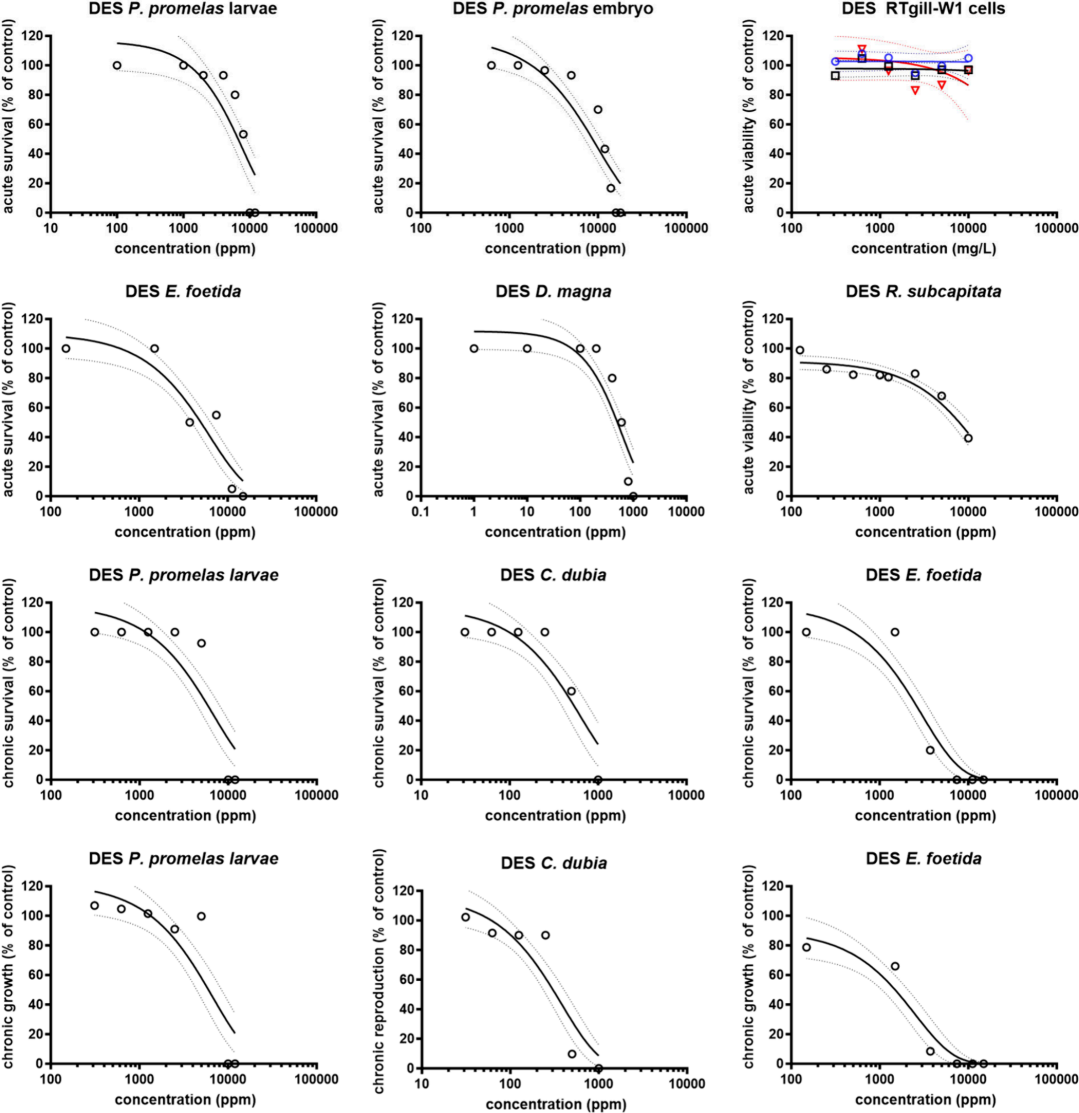
Dose–response curves of DES (ChCl:MgCl_2_·6H_2_O) to model organisms. Results are reported as percent survival based on the control. Values were reported as mean (markers) and confidence intervals (dashed lines) of at least three independent experiments (*n* = 3).

**Figure 2:**
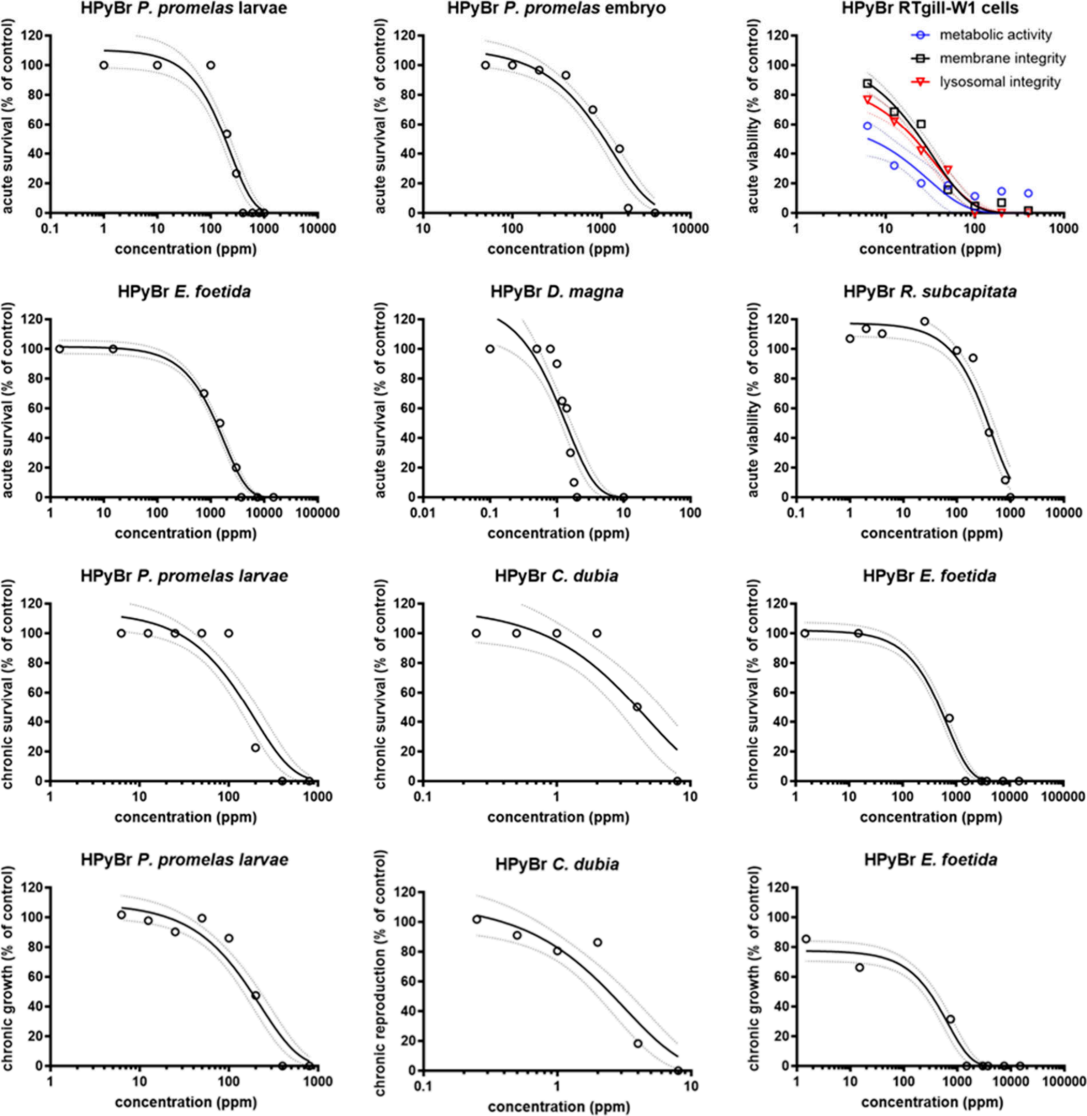
Dose–response curves of HPyBr compared to model organisms. Results are reported as percent survival based on control. Values reported as mean (markers) and confidence intervals (dashed lines) of at least three independent experiments (*n* = 3).

Table 2 summarizes the performed toxicity end point results for HPyBr and ChCl:MgCl_2_·6H_2_O. A summary of the dose–response curves for DES and HPyBr is shown in Figures 1 and 2, respectively.

**Table 2:** Toxciological End Point Values for Model Species

Compound	Exposure type	Duration	Species	End point	Concentration (ppm)
HPyBr	Acute	96 h	*Daphnia magna*	LC50	1.52 ± 0.24
	Chronic	7 d	*Ceriodaphnia dubia*	LC50	3.91 ± 1.5
	Acute	24 h	*Oncorhynchus mykiss* - cells	EC50^a^	13.4 ± 1.65
	Chronic	7 d	*Pimephales promelas* - larvae	LC50	193 ± 3.82
	Acute	48 h	*Pimephales promelas* - larvae	LC50	215 ± 23.6
	Acute	96 h	*Raphidocelis subcapitata*	EC50	382 ± 28.4
	Chronic	14 d	*Eisenia fetida*	LC50	738 ± 17.7
	Acute	96 h	*Pimephales promelas* - embryo	LC50	1151 ± 123
	Acute	7 d	*Eisenia fetida*	LC50	1319 ± 336
					
ChCl:MgCl_2_·6H_2_O	Chronic	7 d	*Ceriodaphnia dubia*	LC50	501 ± 134
	Acute	96 h	*Daphnia magna*	LC50	573 ± 109
	Chronic	14 d	*Eisenia fetida*	LC50	3238 ± 750
	Acute	7 d	*Eisenia fetida*	LC50	5344 ± 1835
	Chronic	7 d	*Pimephales promelas* - larvae	LC50	5888 ± 735
	Acute	96 h	*Raphidocelis subcapitata*	EC50	7900 ± 1459
	Acute	48 h	*Pimephales promelas* - larvae	LC50	7916 ± 455
	Acute	96 h	*Pimephales promelas* - embryo	LC50	11332 ± 536
	Acute	24 h	*Oncorhynchus mykiss* - cells	EC50^a^	NC^b^

a Geometric mean of all three cellular end points, metabolic activity, membrane integrity, and lysosomal integrity.

b Not calculated due to lack of toxicity.

Overall, the results showed that HPyBr toxicity was higher in all species tested for acute and chronic end points compared to ChCl:MgCl_2_·6H_2_O DES (Table 2, Figures 1 and 2). The results indicate that HPyBr and ChCl:MgCl_2_·6H_2_O are the most toxic to daphnid species and the least toxic to the fish embryo. The exact mechanism of toxicity warrants further investigation to determine the mode of action that might be occurring to target specific species. One explanation could be that the chemical structure relates to toxicity. Stock et al.^34^ explored imidazolium based ILs with different side chains and anions using an enzymatic assay method. The toxicity trend was found to vary depending on the type of anion and the alkyl chain length, with an increase in alkyl chain length correlating with higher toxicity of the ILs.

**Figure 3:**
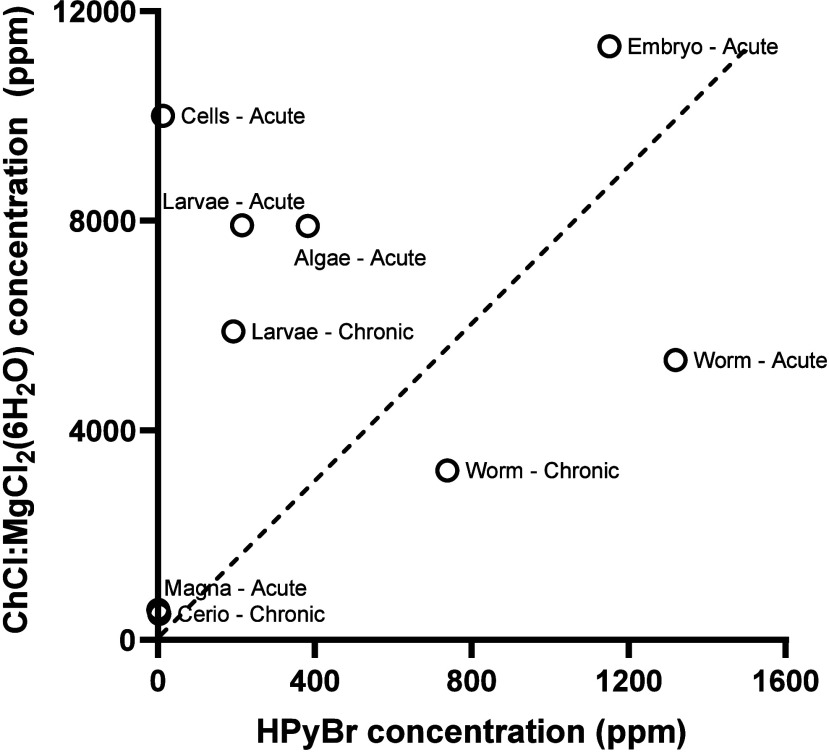
Correlation of toxicity end points for model organisms to HPyBr and ChCl:MgCl_2_·6H_2_O. Dashed line represents the line of unity between HPyBr and ChCl:MgCl_2_·6H_2_O. Effect concentrations 50% of population values (EC50s) are reported for acute toxicity to algae (*Raphidocelis subcapitata*) and RTgill-W1 cells (*Oncorhynchus mykiss*). Lethal concentrations 50% of population (LC50s) are reported for acute toxicity for *Daphnia magna* and *Pimephales promelas* embryo and larvae, chronic toxicity for *Ceriodaphnia dubia*, and acute and chronic toxicity for *P. promelas* larvae and worms (*Eisenia fetida*).

Additionally, Ranke et al.^14^ conducted a Microtox bioassay using *Vibrio fischeri* (V. fischeri) to investigate the impact of alkyl chain length in ILs. They made an imidazolium series with varying alkyl chain lengths (3 to 10 carbon atoms) and fixed anionic structures (tetrafluoroborate [BF4], hexafluorophosphate [PF6], and Chloride [Cl]). The number of alkyl chain atoms exhibited a linear correlation with the EC50 values of the imidazolium-based ILs. Other studies reported similar findings.^15,35−37^ Notably, the findings from the present study have indicated that certain organismal species are more likely to experience harmful toxicological insults from both chemicals (Figure 3), with HPyBr being the most toxic. Therefore, caution should be taken when ILs are used in environmental settings specific to aquatic or soil matrices, for instance, in the case of uncontrolled release in geothermal application.

Studies have shown that many choline chloride based DESs exhibit low cytotoxicity, with EC50 values often exceeding 10 mM in fish and human cell lines.^38^ However, cytotoxicity can vary depending on the hydrogen bond donor, with organic acid–based DESs showing higher toxicity.^39^ While generally nontoxic to bacteria,^40,41^ the cytotoxicity of DESs can be higher than their individual components, suggesting unique toxicological behavior.^40^ Phytotoxicity tests indicate low toxicity, with seed germination EC50 values above 5000 mg·L^–1^.^38^ Most choline chloride-based DESs are readily biodegradable, with high mineralization levels of 68–96%.^38,41^

### 3.1 Algae

Acute toxicity was shown to affect the algal cell growth for both chemicals. Toxicity ranking indicated algae to be similar in the species sensitivity comparison for both chemicals among other species with an EC50 of 350 and 7900 ppm for HPyBr and ChCl:MgCl_2_·6H_2_O, respectively. Latala et al.^42^ studied the effect of the alkyl chain length of ILs using imidazolium cores with varying alkyl chain lengths on marine algae species in the southern Baltic Sea. Their findings showed that a longer alkyl chain is linked to a more potent growth inhibition of the microalgae. The toxicity of bromide-based ILs to algae increases with alkyl chain length, as demonstrated in studies on various freshwater algal species.^43−45^ These ILs can inhibit algal growth, damage cell structures, and impair photosynthetic activity.^45,46^ The LC50 values for different algal species vary widely, ranging from 0.005 to 2138 mg·L^–1^, with Scenedesmus species generally being more sensitive than Chlamydomonas or Chlorella.^43,44^ The toxic effect of ILs was mitigated by high salinity.^42,47^ Additional exploration into the harmful effects of elongating the alkyl chain length on microalgae was performed.^43,48^ As crucial as algal species are to forming an energy base for aquatic food webs, concerns should be raised about the impacts of ILs on their ecological relevance.

Studies have shown that many DESs exhibit low toxicity to various organisms, including bacteria, algae, crustaceans, and fish.^38,49,50^ However, some DESs can cause growth inhibition or biostimulation in algae at certain concentrations.^50^ The toxicity of DESs is influenced by their chemical composition, with choline chloride-based DESs generally showing lower toxicity compared to those based on other quaternary ammonium salts.^38,51^

### 3.2 Daphnia

As mentioned earlier, both daphnia species were observed to be the most sensitive to both chemicals. *D. magna* was shown to be acutely toxic with LC50 values of 1.52 and 573 ppm for HPyBr and ChCl:MgCl_2_·6H_2_O, respectively. Chronic toxicity was measured for *C. dubia* for both mortality with an LC50 of 3.91 and 501 and reproduction with a NOEC of 2 and 250 ppm for HPyBr and ChCl:MgCl_2_·6H_2_O, respectively.

Compared to the literature, Wang et al.^52^ conducted a 48-h acute toxicity assay with *D. magna* of HPyBr and reported a logLC50 of 0.548 mg·L^–1^ and compared his results to Couling et al.^35^ with a logLC50 of 0.457 mg·L^–1^, which are within the results obtained in the study when considering the test duration. From another bromide-based IL study, the 48-h toxicity of [bmim]Br to *D. magna* showed an LC50 of 8.03 mg·L^–1^ indicating that HPyBr is more toxic than [bmim]Br.^53^ The mean 24 and 48 h [C8mim]Br LC50 values for *D. magna* were 1.99 and 0.95 mg·L^–1^ under laboratory conditions.^54^ Bernot et al.^53^ conducted toxicity tests on 1-butyl-3-methylimidazolium [IM14]-based ILs with the anions [Br], [Cl], [PF6], and [BF4], assessing their impacts on the lethality, and reproduction of *D. magna*. The tested ILs’ toxicity was comparable to phenol, trichloromethane, and tetrachloromethane but higher than other organic solvents such as benzene, methanol, and acetonitrile. Therefore, future studies aimed at 21 days chronic *D. magna* tests are warranted to determine if the current ILs in our study pose additional harmful impacts on reproduction. The impacts of zooplankton on aquatic systems are concerning as they play an essential role as an intermediary species responsible for providing a food source from primary producers to larger invertebrate and vertebrate populations.

### 3.3 RTgill-W1 Cells

**Figure 4:**
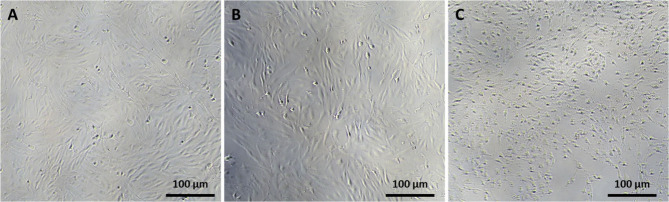
Phase contrast imaging of (A) control RTgill-W1 cells exposed to (B) ChCl:MgCl_2_·6H_2_O and (C) HPyBr at 10,000 ppm.

Cytotoxicity assays for RTgill-W1 cells indicated acute toxicity only for HPyBr with a measured EC50 of 13.4 ppm and were shown to be the second most sensitive model after daphnids. All three subcellular end points were shown to induce a cell viability reduction upon HPyBr exposure. Morphology indicated abnormalities for HPyBr, with the highest concentration being exposed at 10,000 ppm, and no difference was observed for ChCl:MgCl_2_·6H_2_O compared to controls (Figure 4).

Ranke et al.^14^ used mammalian cell culture methods to additionally study the structural impacts of ILs, such as alkyl chain length and types of anions, on the viability of IPC-81 cells. Imidazolium-based ILs with varying alkyl chains (C3–C10) and different anions ([BF4], [PF6], [Cl], and Bromide [Br]) were used. The elongation of alkyl chains intensified the hydrophobicity of the ILs, a factor associated with heightened IL toxicity. Exposure of cells to low concentrations of ILs leads to a hormetic effect, a positive response in the presence of low concentrations of a toxicant, and cell viability.^14,55^ Using the C6 rat cell line test, the effect of alkyl chain length on R1 was found to be highly linked with the log EC50 values of the ILs; however, the impact of anions on toxicity remains unclear.^56^ Notably, the study has shown that the use of piscine cells can be used for measuring toxicity of certain ILs such as HPyBr; however, ChCl:MgCl_2_·6H_2_O was not able to be used to predict toxicity and warrants further investigation as to how the mechanism of toxic action is limited to this specific IL.

### 3.4 Embryos

Fish embryos were shown to have less sensitivity to most species to both chemicals with LC50 values of 1151 and 11,332 ppm for HPyBr and ChCl:MgCl_2_·6H_2_O, respectively. This result may be because the chorion acts as a protective barrier between the embryo and toxicant insults or organic chemicals.^57^ Previous studies have shown that ILs negatively affect the hatching rate of fish embryos.^58^ Studies on goldfish embryos showed that 1-methyl-3-octylimidazolium bromide ([C8mim]Br) caused developmental delays, decreased hatching rates, and increased malformations.^59^ Similar effects were observed in snail embryos, with [C8mim]Br inhibiting hatching and causing deformations at low concentrations.^60^ The 96 h LC50 values of [C8mim]Br on the tested snails at three developmental stages (juvenile, subadult, and adult) were 70.83 ± 2.99, 97.59 ± 4.05, and 109.3 ± 2.22 mg/L, respectively, indicating that young snails were more sensitive to [C8mim]Br toxicity than adults.^60^

**Figure 5:**
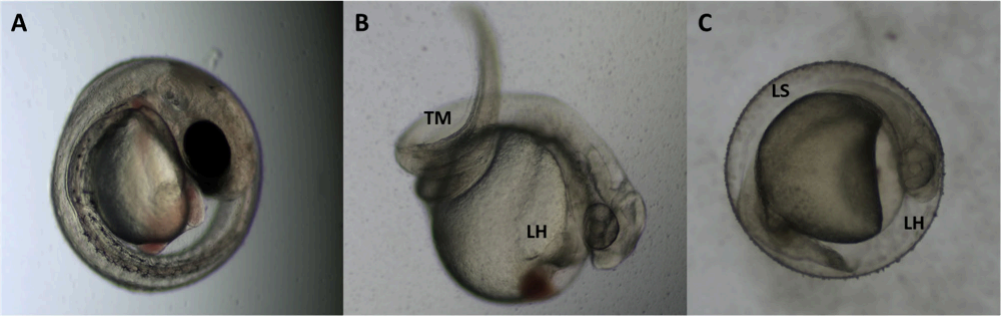
Phase contrast imaging of (A) control fathead minnow (*Pimephales promelas*) embryos exposed to (B) ChCl:MgCl_2_·6H_2_O and (C) HPyBr at 10,000 ppm. Abbreviations indicate tail deformation (TM), lack of somite development (LS), and lack of heartbeat (LH).

Future studies investigating the current ILs’ impact on hatch success are warranted to determine critical embryonic developmental success. Additionally, an extension of the test duration to include eleuthero embryonic stages up to 120-h post fertilization may allow additional teratogenic effects to be measured along with more sensitive end points such as snout length, eye size, pericardial edema, and heart rate.^27,28^Figure 5 shows images of the control and exposed fathead minnow embryo tests.

### 3.5 Fathead Minnow Larvae

Fish larvae assays indicated similar mortality for HPyBr between acute and chronic exposures with LC50 values of 215 and 193 ppm, respectively. Fish growth was affected by HPyBr exposure with an NOEC value of 100 ppm. For ChCl:MgCl_2_·6H_2_O, acute and chronic toxicity were less toxic with LC50 values of 7900 and 5888 ppm, respectively. Previous studies investigated acute toxicity of ILs to fish and found that with an increase in concentration, histopathological changes increased in the gill, skin, liver, kidney, and intestine of zebrafish (*Danio rerio*).^61^

### 3.6 *Eisenia foetida* Earthworms Soil Toxicity

Earthworm assays showed HPyBr exposure to be the least sensitive for acute and chronic toxicity responses compared to the other species, excluding the fish embryos. LC50 values for HPyBr were 1319 and 738 ppm, respectively. However, for ChCl:MgCl_2_·6H_2_O, earthworms were the third most sensitive species with acute and chronic toxicity LC50 values of 5344 and 3288, respectively. Therefore, it is essential to consider the application of each IL for harmful impacts on terrestrial invertebrates, such as earthworms.

Previous studies investigated ILs and their impact on the digestive system of earthworms and found that they may affect protein-related binding functions and catalytic activity (Chu et al., 2023).^62^ However, transcriptome analysis did not identify the mechanism of toxicity using conventional end points. The toxicity of imidazolium-based ILs on earthworms has been investigated in several studies. Acute toxicity tests revealed that LC50 values decreased with increasing alkyl chain length, indicating higher toxicity for longer-chain ILs.^63,64^ However, a cutoff effect with decreasing toxicity was observed for long chains.^63^ Luo et al.^64^ exposed Eisenia Foetida to [C8mim]Br, where the 7-d LC50 was 206.8 mg.kg^–1^ and the 14-d LC50 was 159.4 mg.kg^–1^ in artificial soil. Subchronic exposure to ILs resulted in adverse effects on earthworms, including inhibition of growth and reproductive ability.^65^ ILs also affect biochemical processes in earthworms, such as altering acetylcholinesterase and cellulase activities.^64^

Further studies are needed to understand the current ILs’ role in soil and sediment impacts in deep-enhanced geothermal systems (EGS). Due to the designs of geothermal wells, the possibility of the geothermal working fluid escaping to formation water is improbable; however, there might be a possibility of surface contamination. Further analysis will guide better regulation for the safe use and disposal of such fluids, including the environmentally relevant concentrations.

## 4 Conclusions

The toxicity of hexylepyradinium bromide (HPyBr) IL and a deep eutectic solvent (DES) comprising choline chloride with magnesium chloride hexahydrate (ChCl:MgCl_2_·6H_2_O) was presented. Five test species: an algal species (*R. subcapitata*), the water fleas *C. dubia* and *D. magna*, the fathead minnow (*P. promelas*), and the earthworm *E. fetida*, were used to measure acute and chronic toxicity. Overall, DES showed lower toxicity levels than HPyBr for all five species. Lipophilicity is a primary driver of toxicity, as it is linked to cation and anion arrangement, and research is needed to investigate IL’s toxic mechanism of action. Comparative toxicity analysis revealed that DES, while having lower toxicity, still threatens aquatic life, and the cumulative impacts of such fluids in the environment should be considered. Toxicity tests on *E. fetida* showed concentration-dependent effects for DES and HPyBr, with severe adverse effects on growth at higher concentrations. Ionic liquids made of toxic cations are poorly understood, especially for new and green ILs. Further investigations of environmentally relevant concentrations of such fluids are required before industrial use with specific responsible handling and disposal or recycling guidelines. Toxicity was measured in multiple trophic-level approaches and should warrant concern for their use in industrial and commercial applications.
